# Coronary Computed Tomography Angiography Improving Outcomes in Patients with Chest Pain

**DOI:** 10.1007/s12410-019-9492-6

**Published:** 2019-03-22

**Authors:** Evangelos Tzolos, David E. Newby

**Affiliations:** 1 British Heart Foundation, Centre for Cardiovascular Science, University of Edinburgh, Chancellor’s Building, Edinburgh EH16 4SA, Scotland, UK

**Keywords:** Computed tomography coronary angiography, Coronary artery disease, Exercise electrocardiography, Stress echocardiography, Myocardial perfusion imaging, Cardiac magnetic resonance imaging

## Abstract

**Purpose of Review:**

To provide an overview of recent studies of coronary computed tomography angiography (CCTA) and how it has helped to improve clinical outcomes for patients presenting with chest pain.

**Recent Findings:**

Randomised controlled trials have uniformly demonstrated that the use of CCTA is associated with improvements in patient diagnosis, management and treatments as well as the avoidance of unnecessary invasive coronary angiography. These changes have been associated with consistent reductions in long-term rates of fatal or non-fatal myocardial infarction.

**Summary:**

Major beneficial effects in clinical management and patient outcomes are seen with the use of coronary computed tomography angiography. CCTA might be considered to be the first test of choice for the investigation of coronary heart disease.

## Introduction

Cardiovascular disease and coronary artery disease are the primary causes of morbidity and mortality in developed countries [1–3]. In the past, obstructive coronary disease was largely studied with indirect diagnostic tests that assess cardiac ischaemia. These tests have developed over time from the exercise electrocardiogram to myocardial perfusion imaging with single-photon emission computed tomography, stress echocardiography, positron emission tomography, and magnetic resonance imaging. They have provided important prognostic information and have been the focus of the most recent international guidelines for the investigation of stable chest pain [4, 5]. Although they can demonstrate inducible ischaemia suggestive of obstructive coronary disease, they are unable to detect non-obstructive coronary artery disease, and the use of these tests has not led to improved clinical outcomes within the setting of randomised controlled trials [4–9]. In contrast, coronary computed tomography angiography (CCTA) has the ability to identify patients with obstructive and non-obstructive coronary heart disease with high sensitivity and specificity [10, 11]. In addition in comparison to function imaging, CCTA use has been associated with reduced non-fatal myocardial infarctions and coronary heart disease death [12, 13].

## Evolution of Coronary Computed Tomography Angiography

Coronary computed tomography angiography is being increasingly utilised in clinical practice for evaluating coronary anatomy for obstructive disease and plaque. Computed tomography was first introduced by Sir Godfrey Hounsfield in the 1970 and the first commercial scanner was available at 1972. In the early 1980s, an important advance helps to demonstrate the potential for CT technology to image the moving heart. This was the introduction of the electron beam computed tomography (EBCT) by Douglas Boyd. This technology decreased scan times and improved temporal resolution. For the first time, it was possible to view cardiac contractions and to visualise small structures such as calcium deposits within the walls of the coronary artery. A limitation of EBCT is the spatial resolution due to the slice width of 1.5 mm. An additional major advance in computed tomography imaging came in the early 1990s with the introduction of multidector computed tomography (MDCT) that could rotate 360°. With these advances, spatial resolution improved due to multidetection technology and temporal resolution compromise was reduced since the X-ray beam was able to rotate continuously around the patient as they moved through the scanner [14]. Early multi-detector row computed tomography scanners introduced in 1998 had four detector rings and were capable of half a second gantry rotations. Today’s multidetector computed tomography scanners have up to 320-detector rings, gantry rotation times as low as 270 msec, and in some cases, two x-ray sources, allowing submillimeter resolution to be acquired over very large volumes in a fraction of a second. Faster volume coverage also allowed a sizable reduction in contrast media usage [15].

According to the current European Society of Cardiology guidelines and American College of Cardiology and American Heart Association appropriate-use criteria, CCTA is a level IIa recommendation as an alternative to a stress test for ruling out stable coronary artery disease in patients with low to intermediate pre-test probability [4, 5]. In contrast, the 2016 update to the National Institute for health and Care Excellence (NICE) chest pain guideline (CG95) recommends CCTA as the first-line test for the evaluation of coronary artery disease in stable chest pain pathways [16]. This guideline is based predominantly on the diagnostic precision and cost effectiveness of this strategy compared to invasive coronary angiography.

## Evaluating Patients with Chest Pain–Functional Assessment

Patients attending the emergency department and outpatient department with chest pain account for almost half of all admissions [17]. The initial evaluation includes history taking, physical examination, ECG and clinical biochemistry and is aiming to identify high-risk patients and those with an acute myocardial infarction. If an acute coronary syndrome is excluded, then questions remain if the pain is cardiac or not, and whether the patient has coronary artery disease. These questions have been troubling physicians for decades. The presenting complaint is frequently atypical in nature, and clinicians are faced with the dual task of avoiding unnecessary investigations whilst also ensuring the safe and efficient identification of those individuals with underlying coronary heart disease. To answer these questions, several functional and anatomical tests have been developed throughout the years.

Exercise treadmill test has been the cornerstone method for evaluating patients with stable chest pain for several decades. Depending on the results, the patient would often receive either medical therapy or be referred for invasive angiography. Although this test is cheap and cost-effective, its sensitivity and specificity remain low (61% and 70%, respectively), and it is even lower in women [18, 19] and lower than other functional imaging modalities [20] leading to unnecessary invasive angiograms or to undertreatment of patients with unrecognised and undiagnosed coronary artery disease (CAPP McKavanagh et al.).

The sensitivity and specificity of functional imaging tests have been reviewed extensively in multiple systematic reviews and meta-analyses (Table 1). Exercise and pharmacological stress echocardiography [21, 22], exercise and pharmacological stress nuclear myocardial perfusion imaging (MPI) [21, 23–25] and pharmacological stress cardiovascular magnetic resonance (CMR) [26, 27] demonstrate an association between abnormal test results and the detection of obstructive coronary artery disease on invasive angiography, as well as an increased risk of adverse cardiovascular events. Randomised controlled trials of functional stress tests that have, however, failed to demonstrate better downstream clinical outcomes in comparison to CCTA (PROMISE, CAPP).

## Evaluating Patients with Chest Pain–Anatomical Assessment

**Fig. 1:**
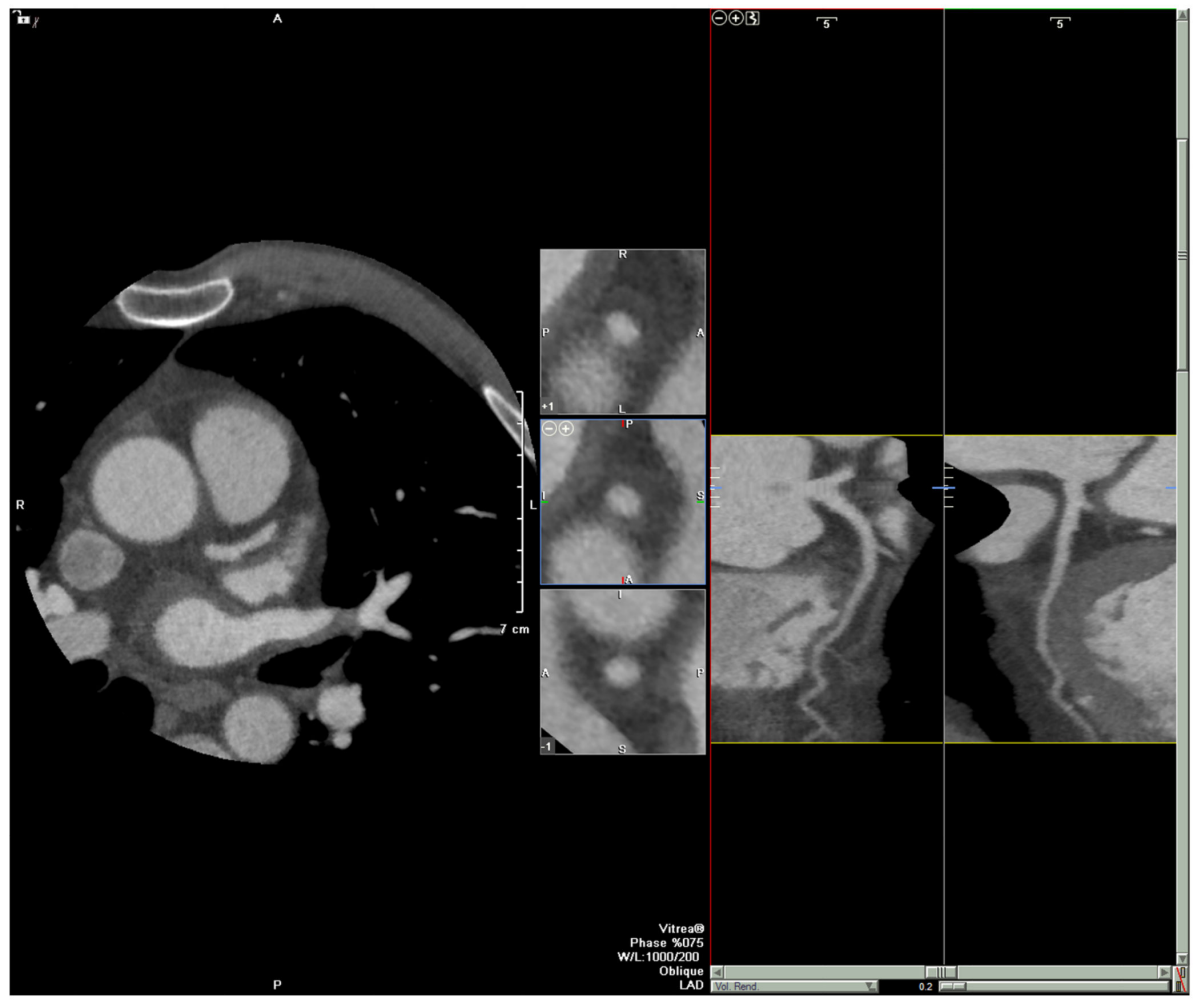
Patient with typical anginal symptoms listed for invasive angiogram following baseline clinical assessment in the SCOT-HEART trial. Normal coronary arteries on coronary computed tomography angiography. Management changes after coronary computed tomography angiography and was treated conservatively. Patients assigned to the coronary computed tomography angiography arm had a reduced likelihood of demonstrating normal coronary arteries in the invasive angiogram (P < 0.001) hazards ratio 0.392 (95% CI, 0.227– 0.676) (Reprinted from Williams et al. JACC 2016;67:1759–1768 under terms of CC BY 4.0)

**Fig. 2:**
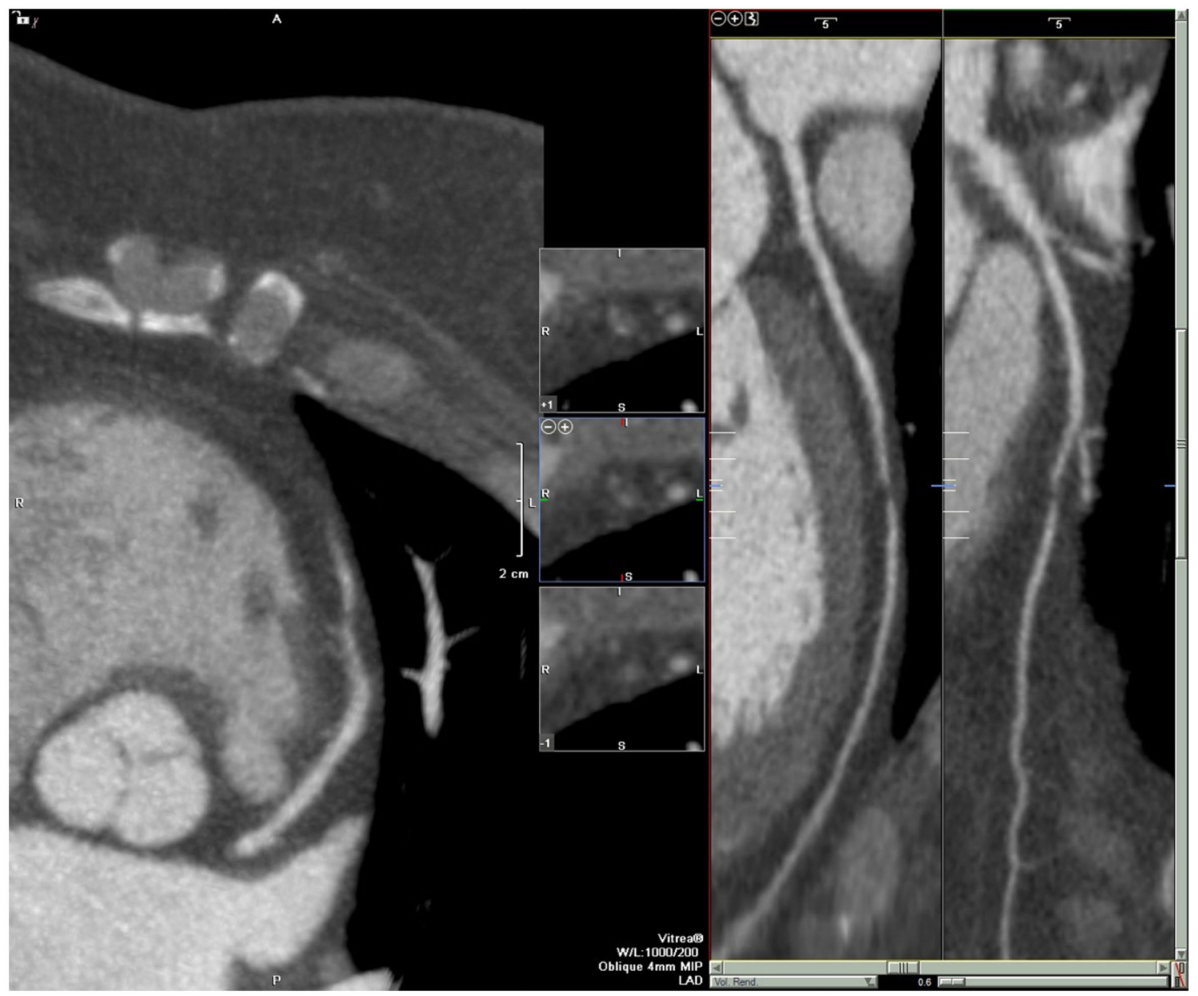
Patient in the SCOT-HEART trial who presented with atypical non-anginal chest pain and was managed conservatively at the baseline clinic assessment. Obstructive coronary artery disease identified on coronary computed tomography angiography. Patients assigned to the coronary computed tomography angiography arm had an increased likelihood of identifying obstructive coronary artery disease in invasive angiogram (P = 0.005), hazards ratio 1.293 (95% CI, 1.081–1.548) (Reprinted from Williams et al. JACC 2016;67:1759–1768 under terms of CC BY 4.0)

With the 64-slice detector computed tomography becoming the minimum standard, the improved temporal and spatial resolution allowed for a high degree of image quality assessment of coronary arteries with CCTA. The extent and severity of angiographic coronary artery disease are amongst the most important prognostic factors and remain vital determinants for revascularisation decision making [28]. Several meta-analyses and clinical trials have reported the diagnostic accuracy of CCTA with 64-slice computed tomography, with sensitivity ranging from 93 to 97% and specificity varying from 90 to 96% [8, 11, 29–33] for identifying obstructive coronary artery disease. Its very high negative predictive value can reassure clinicians and of course give patients peace of mind (CAPP and Scot Heart)—this is extending the CAPP findings that CT reduced ER visits in comparison to treadmill testing. Williams et al. [32] showed that following a strategy of CCTA versus usual care, subsequent clinically requested invasive coronary angiography was less likely to demonstrate normal coronary arteries in the CCTA arm in comparison to usual care (20 vs. 56, respectively; HR: 0.39 [95% CI: 0.23 to 0.68]; p < 0.001) and more likely to show obstructive coronary artery disease(283vs.230,respectively; HR: 1.29 [95% CI: 1.08 to 1.55]; p = 0.005) in the Scottish COmputed Tomography of the HEART (SCOT-HEART) trial (Figs. 1 and 2). In this study, the authors demonstrated that the addition of CCTA to routine clinical assessment of patients with suspected angina pectoris secondary to coronary heart disease leads to a nearly 3-foldr eduction in the rates of normal invasive coronary angiography.

**Fig. 3:**
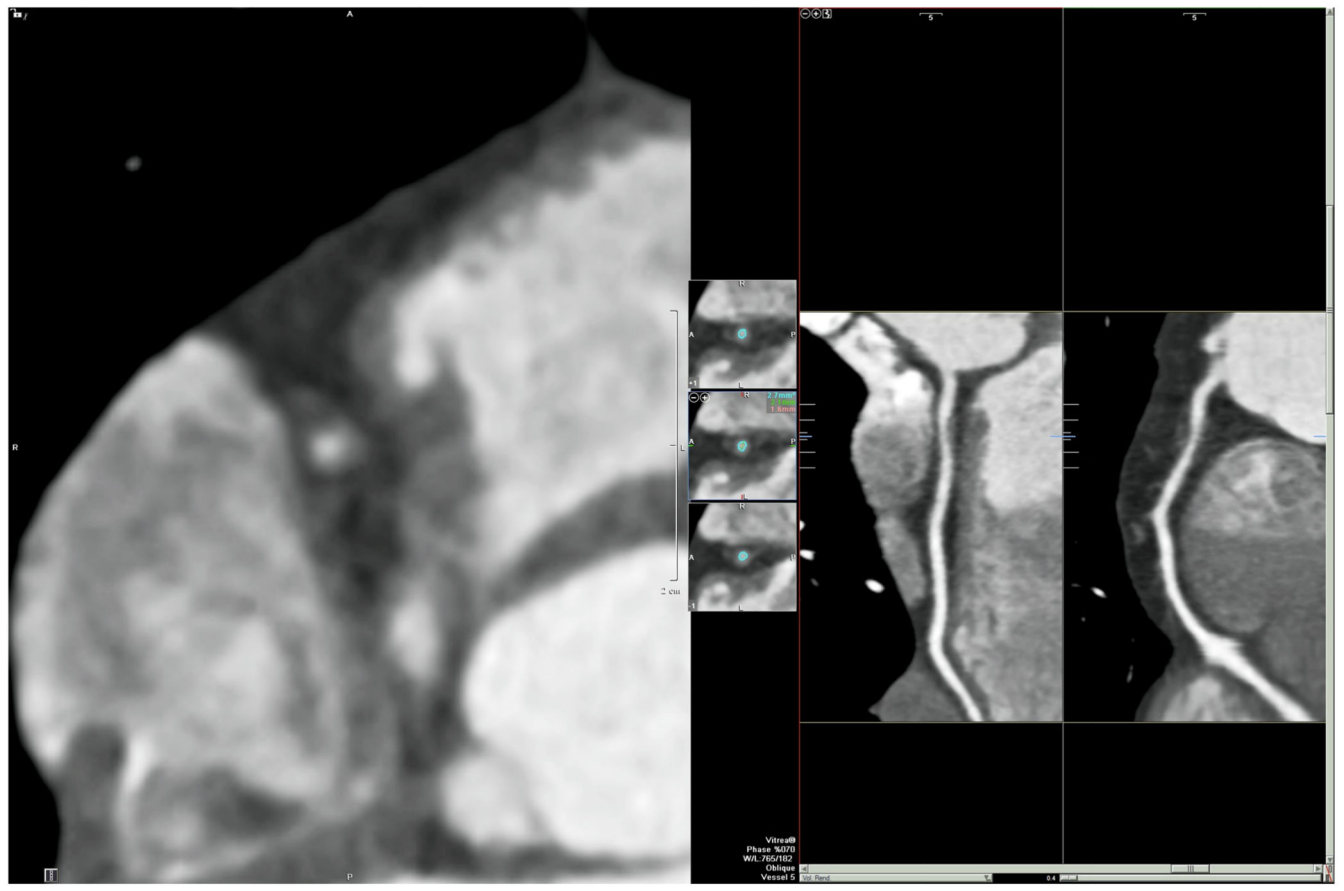
A 47-year-old man presented with atypical chest pain and was found to have significant non-obstructive proximal right coronary artery disease and a calcium score of zero

In addition to precisely detecting obstructive coronary artery disease [34], CCTA provides prognostic information related to the presence and extent of non-obstructive plaque [35]. Since the incidence of nonobstructive plaque is more likely to be recognised by CCTA, it is associated with a higher use of downstream preventive therapies and better risk factor control, therefore leading to improved outcomes [36–38]. CCTA also reliably illustrates the morphology and composition of coronary atherosclerosis, including high-risk plaque features, such as positive remodelling and low attenuation plaque disease (Fig. 3).

## Asymptomatic Individuals

The FACTOR-64 trial [39] has been the only CCTA trial in primary prevention, and it specifically recruited 900 patients with type 1 or 2 diabetes mellitus only. Participants found to have coronary heart disease on computed tomography coronary angiography were targeted for more intensive risk factor modification although 75% of trial participants were already on a statin at baseline. Compared to standard of care, those assigned to CCTA had an LDL-cholesterol concentration that was 0.06 mmol/L lower (p = 0.02), but there was no difference in blood pressure or haemoglobin A1c concentrations. In the intention-to-treat analysis, the primary endpoint occurred in 6.2% of the CCTA group compared to 7.6% in the control group (hazard ratio, 0.80 [95% confidence interval, 0.49–1.32]; p = 0.38). In the as-treated analysis, the respective event rates were 5.6% vs 7.9% (hazard ratio, 0.69 [95% confidence interval, 0.41–1.16]; p = 0.16). The failure to demonstrate a benefit is therefore likely to represent the inability to deliver a major difference in treatment and management consequent on the application of the imaging test and a lack of power due to the small sample size and lower than anticipated event rate.

## Patients with Stable Chest Pain

Several studies have demonstrated improved outcomes when CCTA is added to standard care in patients with stable chest pain (Table 2). Recently, our group reported the 5-year outcomes of the SCOT-HEART trial where we identified a 41% reduction in the composite endpoint of coronary heart disease death or non-fatal myocardial infarction amongst participants randomised to the CCTA in addition to standard of care [12]. This observed lower rate of the primary clinical end point was driven mainly by a lower rate of non-fatal myocardial infarction.

Although in the first 12 months, there was an increase in the invasive angiograms and coronary revascularisation performed in the CCTA group, there was no difference between the two groups in terms of invasive angiography or coronary revascularisation by 5 years of follow-up [12]. This is consistent with better and earlier identification of disease that led to more appropriate invasive angiography and coronary revascularisation in the first year. Indeed, other trials with short-term follow-up have also shown higher rates of invasive coronary angiography and coronary revascularisation after coronary computed tomography angiography than after functional testing [40]. However, beyond 1 year in the SCOT-HEART trial, the prior increase in revascularisation appeared to pay dividends because the rates of invasive angiography and coronary revascularisation became lower than the standard of care group suggesting progression of untreated unrecognised disease in those who had not undergone a CCTA. Whilst it is plausible that early revascularisation played a role in the observed long-term difference in events, the benefits are likely to be mostly attributable to changes in medical management.

The benefits of preventative medical therapy are well described in major randomised controlled trials. Nevertheless, it should be remembered that many trials (particularly primary prevention trials) treated a general and broad population of patients at risk of cardiovascular disease. Most of these trial participants did not have cardiovascular disease and they had no chance of benefiting from the intervention. In SCOT-HEART, we identified a population with the disease before treatment initiation, which potentially led to greater proportionate benefits. Similarly, in the JUPITER trial, patients were risk stratified according to elevated high-sensitive c-reactive protein, enabling the identification of a high-risk population who then received a more marked benefit from rosuvastatin (hazard ratio, 0.56 (95% confidence interval, 0.46 to 0.69), P < 0.001) than has been seen in previous primary prevention trials [41].

The findings from the SCOT-HEART trial are supported by two other recent studies reviewing patients with stable angina. The CAPP [42] (cardiac computed tomography for the Assessment of Pain and Plaque) and CRESCENT [43] (Computed Tomography vs. Exercise Testing in Suspected Coronary Artery Disease) trials randomised patients to CCTA or either exercise electrocardiography or stress echo-cardiography with approximately 1 year of follow-up. Both trials showed an increased diagnosis of coronary heart disease and consequently increased use of preventative medical therapies in those allocated to CCTA. In addition, despite being underpowered for clinical events, both trials demonstrated lower numerical rates of myocardial infarction amongst those assigned to CCTA. The CRESCENT trial also showed that after CCTA, the final diagnosis was established sooner (P < 0.0001), and additional downstream testing was required less frequently compared to functional assessment (25 vs. 53%, P < 0.0001), resulting in lower cumulative diagnostic costs [43].

To add to the above, the Prospective Multicenter Imaging Study for the Evaluation of Chest Pain (PROMISE) showed that, although there was no difference in the overall primary outcome, CCTA predicted subsequent cardiovascular events better than functional testing [13, 44]. In the CCTAgroup, the majority of events occurred amongst subjects with non-obstructive coronary artery disease: disease that would pass undetected by functional testing and would be less likely to be associated with initiation of preventative therapy. The investigators also reported that in the CCTA arm, there was a 34% relative reduction in all-cause death and myocardial infarction at 12 months in comparison to functional testing (hazard ratio 0.66 (95% confidence intervals, 0.44-1.00), P = 0.049). Although more patients in the CCTA arm underwent invasive angiography within the first 90 days, fewer invasive angiograms without obstructive coronary artery disease were seen in the CCTA group compared to those who had initial functional testing.

In addition to these individual studies, a meta-analysis of randomised controlled trials published in 2016 comparing CCTA with standard care identified an incidence rate ratio for myocardial infarction of 0.69 following CCTA (95% CI 0.49-0.98;p = 0.038) [45]: aresult entirely consistent with the SCOT-HEART and PROMISE findings, and confirmed in two subsequent larger meta-analyses by independent groups [46,47]. Finally, reductions in myocardial infarction have also been reported in a very large *(n =* 86,705) observational Danish registry (HR for CCTA: 0.71, 95% CI 0.61 to 0.82) [48], demonstrating consistency within ‘real world’ practice.

## Patients with Acute Chest Pain

There have been several studies assessing the value of CCTA in the emergency department using a surrogate of early and safe discharge (Table 3). The largest of them, the American College of Radiology Imaging Network–Pennsylvania trial (ACRIN-PA) [49], randomised 1370 low- to intermediate-risk patients presenting to five emergency departments with symptoms suggestive of acute coronary syndrome to either CCTA or traditional chest-pain care. The trial found that CCTA allowed more patients to be discharged safely than standard of care (49.6% vs 22.7%), and this led to a shorter average hospital stay (18 h versus 25 h, p < 0.001). Coronary artery disease detection rate was also higher in the CCTA group (9% vs 3.5%) allowing for greater initiation of secondary preventative treatment. Similar results were observed in the ROMICAT-II trial [50] which included 1000 patients aged 40 to 74 years with symptoms suggestive of acute coronary syndromes, no history of cardiovascular disease and initial testing (electrocardiogram and troponin measurements) did not clearly indicate a myocardial infarction. Mean length of hospital stay was reduced by 7.6 h (P < 0.001) after early CCTA, as compared with standard of care. Additionally, more patients assigned CCTA were discharged directly from the emergency department (47% vs. 12%; P < 0.001). In the Cardiac-CT in the Treatment of Acute Chest Pain (CATCH) trial, a CCTA-guided diagnostic strategy improved the positive predictive value for the detection of coronary stenoses and increased the frequency of coronary revascularisation when compared to a conventional functional approach [51]. Similarly, Goldstein et al. in Coronary Computed Tomographic Angiography for Systematic Triage of Acute Chest Pain Patients to Treatment (CT-STAT) study showed that the use of coronary computed tomography angiography results in more rapid and cost-efficient safe diagnosis than rest-stress myocardial perfusion imaging in patients with acute low-risk chest pain [52].

A meta-analysis by Gongora et al. [53] showed that CCTA improves efficiency measures in the acute care settings. It failed to show reduction in major adverse cardiac events in patients presenting to the emergency room or admitted for acute chest pain evaluation but the overall adverse cardiac event rates were very low since the studies recruited low-risk and low-to-intermediate risk patients. In this regard, it will be interesting to see the results of the RAPIC-CTCA trial [54] which will provide more valuable information regarding the reduction of MI or cardiovascular death with coronary computed tomography angiography in the acute setting as it will recruit 2000 higher-risk participants across 35 hospitals in the United Kingdom. It is the first study to investigate the role of CCTA in the early assessment of patients with suspected or confirmed acute coronary syndrome who are at intermediate risk, including patients with elevated troponin concentrations or ischaemic changes on the electrocardiogram. All previous trials in the emergency department have enrolled patients who are at low risk of acute coronary syndrome, supported by the exceptionally low subsequent 30-day and 1-year reported outcomes.

## Conclusions

Recent studies as well as large meta-analyses have demonstrated that the use of CCTA is associated with important reductions in coronary heart disease death or non-fatal myocardial infarction [55]. It also provides precise disease characterisation and reduces the rate of normal invasive coronary angiography. The more universal message from these trials is that the information provided by a diagnostic test can reverberate therapeutically beyond making a correct diagnosis of coronary artery disease and that clinicians should pursue preventive measures to achieve the best outcomes possible. Ultimately, the improved diagnosis and treatment of coronary heart disease coupled together with the treatment of concealed non-obstructive coronary artery disease underlie and explain the important beneficial effects of CCTA.

## Figures and Tables

**Table 1:** Sensitivity and specificity of different functional assessments for obstructive coronary artery disease

Functional test	Sensitivity	Specificity
Exercise electrocardiography	61%	70%
Exercise stress echocardiography	70–85%	77–89%
Pharmacological stress echocardiography	85–90%	75–90%
Exercise stress SPECT	82–88%	70–88%
Pharmacological stress SPECT	88–91%	75–90%
Dobutamine cardiac magnetic resonance	83%	86%
Adenosine cardiac magnetic resonance	91%	81%

*SPECT* single-photon emission computed tomography

**Table 2:** Coronary computed tomography angiography in stable chest pain (^*^FACTOR-64 is a primary prevention study)

Trial	Intervention arm	Comparator arm	Primary end point(s)	Follow-up, month
Minetal., 2012	Coronary computed tomography angiography(n=91)	Myocardial perfusion imaging 100%(n=89)	Near-term angina-specific health status	2
Douglas et al. (PROMISE), 2015	Coronary computed tomography angiography(n=4996)	Myocardial perfusion imaging, 67%; stress echocardiography, 23%; Exercise electrocardiography,10% (n = 5007)	Composite of death, myocardial infarction, hospitalisation for unstable angina,ormajor procedural complication	25
SCOT-HEART, 2015	Coronary computed tomography angiography and standard of care(n=2073)	Standard of care (n=2073)	Certainty of angina due to coronary heart disease at 6 weeks	20
CAPP, 2015	Coronary computed tomography angiography (n=243)	ETT,100%(n=243)	Changeinangina score from baseline to 3 months	12
FACTOR-64^*^ (^*^Primary prevention)	Coronary artery disease screening with coronary computed tomography angiography(n=452)	Standard national guidelines-based optimal diabetes care(n=448)	all-cause mortality, non-fatal myocardial infarction, or unstable angina requiring hospitalisation	48

**Table 3:** Coronary computed tomography angiography in patients presenting with acute chest pain

Trial	Intervention arm	Comparator arm	Primary end point(s)	Follow-up, month
Goldstein et al., 2007	Coronary computed tomography angiography with MPI for all indeterminate stenoses (*n* = 99)	Myocardial perfusion imaging, 100%(*n* = 98)	Not specified	6
Goldstein et al. (CT-STAT), 2011	Coronary computed tomography angiography with MPI for all indeterminate stenoses (*n* = 301)	Myocardial perfusion imaging, 100% (*n* = 338)	Time to diagnosis	6
Miller et al., 2011	Coronary computed tomography angiography (n = 30)	Not specified (n = 30)	Total resource use	3
ACRIN/PA, 2012	Coronary computed tomography angiography (*n* = 908)	Stress test with imaging, 56%; exercise electrocardiography, 2%; no test, 42% (*n* = 402)	Absence of myocardial infarction and cardiac death during first 30 days in subgroup with negative coronary computed tomography angiography	1
Hoffman et al. (ROMICAT-II), 2012	Coronary computed tomography angiography (*n* = 501)	Myocardial perfusion imaging, 25%; stress echocardiography, 20%; exercise electrocardiography, 29%; no test, 26% (n = 499)	Length of hospital stay	1
Linde et al. (CATCH), 2013	Coronary computed tomography angiography (*n* = 285)	EBT,76%; Myocardial perfusion imaging, 22% (*n* = 291)	Referral rate for invasive coronary angiography, positive predictive value for coronary artery disease and subsequent revasculirisations	4
Hamilton-Craig et al. (CT-COMPARE), 2014	Coronary computed tomography angiography (*n* = 322)	exercise electrocardiography, 100% (=240)	Diagnostic performance for acute coronary syndrome	12
PROSPECT, 2015	Coronary computed tomography angiography (*n* = 2000	Myocardial perfusion imaging, 100% (*n* = 200)	Cardiac catheterization not leading to revascularisation	12
Uretsky et al. (PERFECT), 2016	Coronary computed tomography angiography (206)	Stress echocardiograph, 88%; Myocardial perfusion imaging, 4% (*n* = 205)	No difference found in time to discharge, change in medication use, downstream testing, and cardiovascular	12
